# Integrated Multipoint-Laser Endoscopic Airway Measurements by Transoral Approach

**DOI:** 10.1155/2016/6838697

**Published:** 2016-02-15

**Authors:** Marie Neitsch, Iris-Susanne Horn, Mathias Hofer, Andreas Dietz, Miloš Fischer

**Affiliations:** Clinic of Otolaryngology, Head and Neck Surgery, Department of Head Medicine and Oral Health, University Hospital Leipzig, Liebigstrasse 12, 04103 Leipzig, Germany

## Abstract

*Objectives*. Optical and technical characteristics usually do not allow objective endoscopic distance measurements. So far no standardized method for endoscopic distance measurement is available. The aim of this study was to evaluate the feasibility and accuracy of transoral airway measurements with a multipoint-laser endoscope.* Methods.* The semirigid endoscope includes a multipoint laser measurement system that projects 49 laser points (wavelength 639 nm, power < 5 mW) into the optical axis of the endoscopic view. Distances, areas, and depths can be measured in real-time. Transoral endoscopic airway measurements were performed on nine human cadavers, which were correlated with CT measurements.* Results.* The preliminary experiment showed an optimum distance between the endoscope tip and the object of 5 to 6 cm. There was a mean measurement error of 3.26% ± 2.53%. A Spearman correlation coefficient of 0.95 (*p* = 0.01) was calculated for the laryngeal measurements and of 0.93 (*p* < 0.01) for the tracheal measurements compared to the CT. Using the Bland-Altman-Plot, the 95% limits of agreement for the laryngeal measurements were satisfactory: −0.76 and 0.93.* Conclusions.* Integrated multipoint-laser endoscopic measurement is a promising technical supplement, with potential use in diagnostic endoscopy and transoral endoscopic surgery in daily practice.

## 1. Introduction

Today, endoscopy of the upper and lower airway with rigid or flexible endoscopes is a standard ENT examination. The qualitative assessment of the macroscopic anatomy is possible due to wide-angle views in long distance and object magnification in short distance [1]. Nevertheless, on-screen based quantitative or morphometric measurements are usually not possible. The lack of the correlation to the object size is determined by the incalculable distance of the endoscope tip to the object and visual distortion at the endoscopic image edges. Different techniques have been described to solve this problem. Sharma et al. described a specific measuring stick which has to be applied during endoscopy. However, it was used for rigid suspension laryngoscopy only [2]. The use of reference bodies for the digital postprocessing of endoscopic images is based on a similar principle. Scales with known distances between predefined points on a reference body are used for image calibration. Recently, specific software algorithms for endoscopic image postprocessing, for example, Visual Simultaneous Localization and Mapping (VSLM) or Endoscopic Lesion Measurement System (ELMS), were published by several authors [3–5]. So far, the specified measurement errors of the described systems do not allow their safe use in daily practice. The development of stereo endoscopes providing 3D visualization may be another approach. Those endoscopes have been introduced in laparoscopic surgery or endoscopy of the gastrointestinal tract [6, 7]. However, the wide diameter of these endoscopes due to the dual-channel technique does not allow their unrestricted use for upper airway evaluation in patients [8].

One of the scientific approaches for endoscopic measurement is based on the principle of photometric stereo due to reflected light radiation depending on the image change caused by endoscope movement. Recovering a 3D shape from endoscopic images has been described assuming specific reflectance characteristics and object extraction from the endoscopic image [9]. The underlying Vogel-Breuß-Weickert (VBW) or Radial Basis Function Neural Network (RBF-NN) models require the acquisition of two endoscopic images with a known *z*-coordinate of the endoscope movement [10, 11]. So far, theoretical approaches have been described using artificial bodies with ideal reflectance characteristics by computer simulation or evaluating small-sized, single polyps during gastrointestinal endoscopy only [11, 12].

Another endoscopic measurement approach is based on the triangulation of laser beams in correlation with the optical axis of the endoscope camera. The determined distance of two perpendicular laser beams projected into the optical axis of an endoscope was used to describe endolaryngeal morphometry [13, 14]. A laser fiber projecting a perpendicular laser beam onto the tracheal wall was used for three-dimensional reconstruction of the tracheas to describe the extent of subglottic and tracheal stenoses [15, 16]. Nakatani et al. modified a commercially available flexible endoscope for the gastrointestinal tract by integrating four laser beams parallel to the optical axis of the camera [1]. However, all of these devices were prototypes which did not become commercially available. Thus, there is still a lack of endoscopic measurement standards in routine clinical practice.

Due to technical development over the last decade transoral laser microsurgery (TLM) and transoral endoscopic robotic surgery (TORS) gained high popularity and drew attention towards transoral surgery as a remarkable paradigm shift in head and neck surgery [17–19]. Endoscopic visualization of the oropharynx, larynx, or hypopharynx in combination with miniaturized robotic instruments allows the precise removal of a tumor resulting in reduced surgery related morbidity as well as noninferior outcome compared to traditional open surgery [20]. The exact descriptions of the extent of a tumor and the tumor size are required for accurate surgical planning. However, given the small size of TORS systems, there is a lack of suitable measuring instruments regardless of the fact that stereo endoscopes with a wide diameter have to be used (12 mm: da Vinci® telemanipulator, Intuitive Surgical Inc., Mountain View, CA, USA).

The aim of this study was to evaluate the feasibility and accuracy of transoral airway measurements with an integrated, multipoint-laser endoscope in real-time.

## 2. Materials and Methods

### 2.1. Multipoint-Laser Endoscope

**Figure 1:**
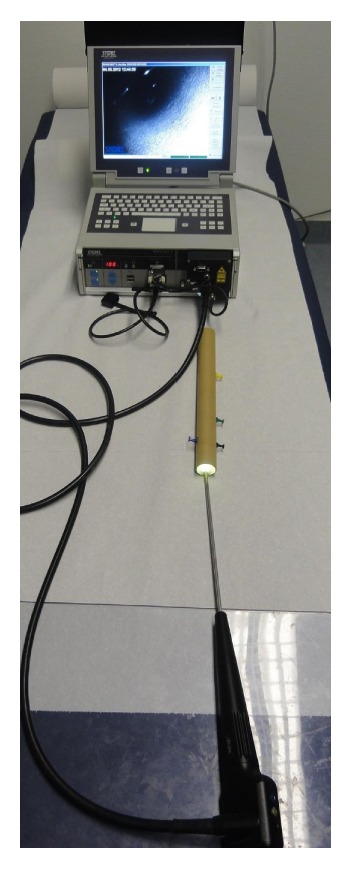
Semirigid endoscope with an integrated multipoint-laser measurement device including a base unit with a LCD flat screen.

**Figure 2:**
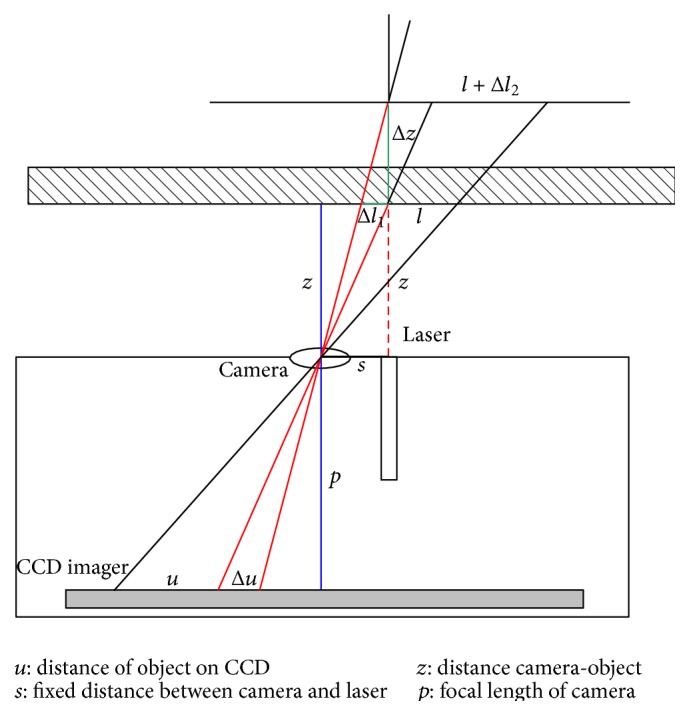
Scheme of laser beam point reflection and detection by the endoscope camera: Δ*u* on the CCD translates to the Δ*l* of the measuring length *l*, by simple geometry.

All measurements were performed with a semirigid endoscope with an integrated multipoint-laser measurement device (Techno Pack® X, Karl Storz GmbH & Co. KG, Tuttlingen, Germany). The system includes a base unit with a LCD flat screen and a modified fiber endoscope in a semirigid sheath with a light source, which is connected to the base unit (Figure 1). The electric products of the Techno Pack X are CE approved. The endoscope includes a multipoint-laser measurement system that projects 49 laser beam points (wavelength 639 nm, power < 5 mW) into the optical axis of the endoscopic view. The prism of the laser and the CCD imager are inside the distal end of the endoscope with a fixed distance. The lateral offsetting of 7 by 7 laser beams from the endoscope pupil forms the basis for the triangular multipoint measurement. Knowing the coordinates of each laser beam point of the laser grid in correlation with the endoscope tip recognized by the CCD imager, the software analyzes the position of each laser beam point depending on the reflection from the surface structure (Figure 2). By generating a three-dimensional model of the object surface in the endoscopic view, distances, areas, and depth can be measured in real-time [21]. At least three laser beam points are required to perform a measurement. If certain laser beam points are not required for a specific measurement, they can be deselected. A calibration of the system prior to each measurement is not necessary.

### 2.2. The Mathematical Principle of Multipoint-Laser Measurement

Formula (1) is as follows:

(1) $$\begin{array}{c} \Delta l=\frac{z}{p}\Delta u_{1}+\frac{z\Delta u_{2}}{sp-z\Delta u_{2}}l=\Delta l_{1}+\Delta l_{2}. \end{array}$$

*Derivation of Formula (1)*. From Figure 2 two basic equations can be derived by symmetry and proportional relations.

Equation a is

(a) $$\begin{array}{c} \frac{\Delta u}{\Delta l_{1}}=\frac{p}{z}. \end{array}$$

Equation b is

(b) $$\begin{array}{c} \frac{\Delta z}{\Delta l_{1}}=\frac{\Delta z+z}{s}. \end{array}$$

Equation a delivers

(1a) $$\begin{array}{c} \Delta l_{1}=\frac{z}{p}\Delta u_{1}. \end{array}$$

This is the first component to formula (1).

Using a different value *u*_2_ now and combining a and b deliver

(c) $$\begin{array}{c} \Delta z=\frac{z^{2}\Delta u_{2}}{sp-z\Delta u_{2}}. \end{array}$$

As the measuring length *l* is proportional to distance *z*, a new d can be set up as

(d) $$\begin{array}{c} \frac{\Delta l_{2}}{l}=\frac{\Delta z}{z}. \end{array}$$

Inserting this in c yields

(1b) $$\begin{array}{c} \Delta l_{2}=\frac{z\Delta u_{2}}{sp-z\Delta u_{2}}l. \end{array}$$

This is the second component in formula (1).

### 2.3. Validation of the Measurement Accuracy

**Figure 3:**
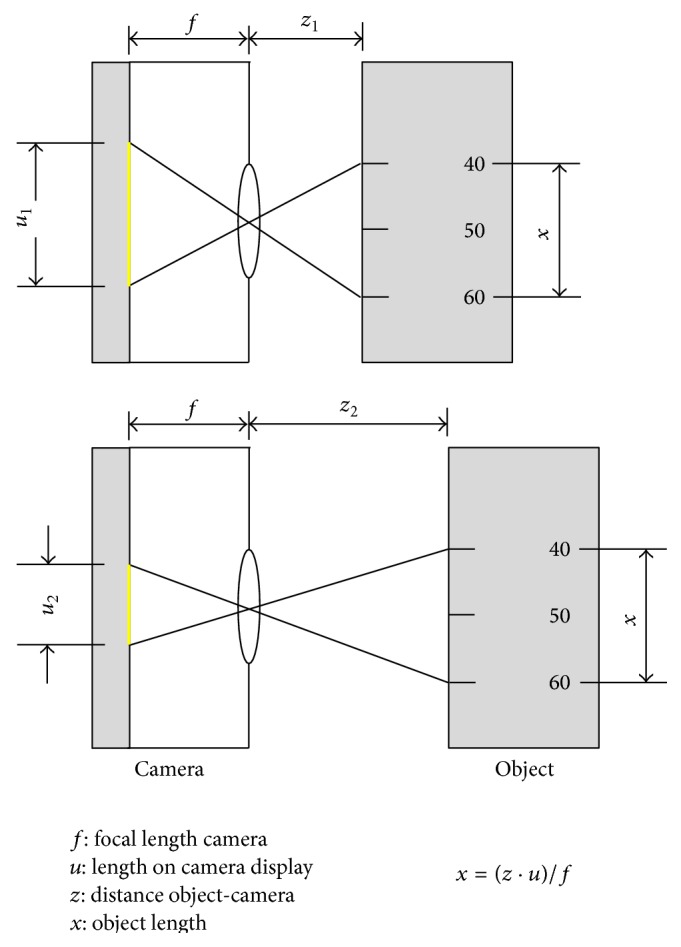
Scheme and mathematical description of measurement evaluation depending on the distance from the endoscope tip.

A laboratory trial was performed as a preliminary experiment to validate the accuracy of the measurement method. An artificial cylindrical hollow body with a length of 30 cm and an inner diameter of 27 mm was used. The endoscope tip was placed at the orifice of the tube. Assuming that the measurement error depends on the distance between camera and object, ten measurements of the tube diameter were taken per cm at distances of 1 to 10 cm from the endoscope tip (Figure 3). The distance from the endoscope tip is equated with the depth of the tube. The distances were marked by pins inside the tube. In addition, a distinction was made between value *A* and value *B* for the measured values obtained for the diameter. This was followed by selection or deselection of the recognized laser beam points to calculate the distance according to different criteria. Value *B* was determined with all recognized laser beam points with a depth of the *z*-coordinate in the same depth as the designated area to be measured (±5 mm). This area was identified as the reference range. In contrast, value *A* was determined from only three recognized laser beam points with a depth of the *z*-coordinate corresponding to the depth of the diameter to be measured or with the smallest deviation from it. This difference was used to verify the dependence of the measurement results on the selection of recognized laser beam points.

**Figure 4:**
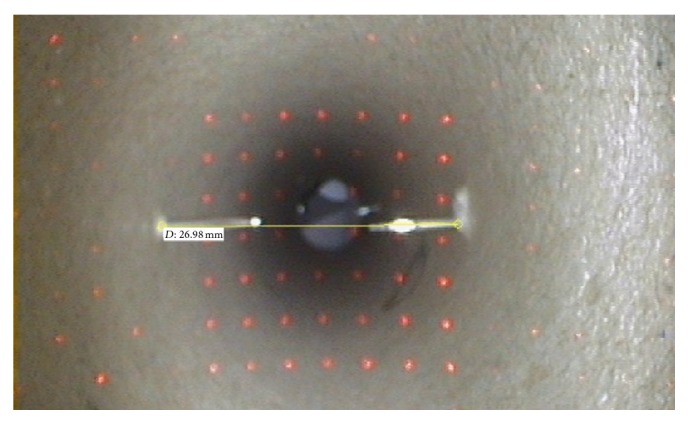
Endoscopic view with distance measurement during validation experiment on a tube model.

In a second experiment, 100 consecutive diameter measurements were performed at a distance of 5 cm from the endoscope tip (Figure 4). The measured values were obtained using all recognized laser beam points with a *z*-coordinate within the reference range (45 to 55 mm). In addition, the number of reference points found was documented.

### 2.4. Measurements on Cadaveric Human Upper Airways

**Figure 5:**
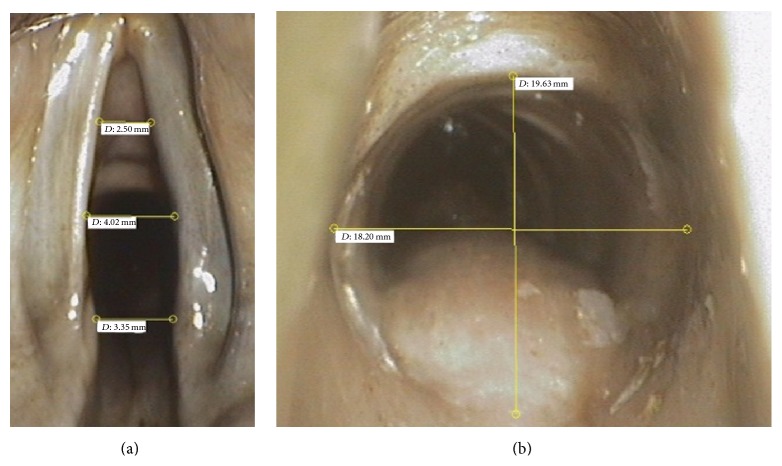
Endoscopic view with distance measurements: (a) laryngeal measurements and (b) tracheal measurements.

Following the preliminary tube examinations, measurements were performed on nine human cadavers, but only the measurements from seven cadavers could be evaluated. The cadavers were Thiel-fixated. Using this fixation method the surface condition and tissue tension are very close to regular tissue. The laser endoscope was used to take transoral measurements in the larynx and the trachea. For this, the distance between the vocal cords in the front third, the middle, and the rear third was measured. The diameter of the trachea was measured at the level of the laryngotracheal junction (Figure 5). In addition, a CT scan of the neck was performed on all the cadavers. The DICOM data were used to measure the corresponding distances in the CT and compared with the measurement values of the laser endoscopy. The CT was considered as the baseline measurement.

### 2.5. Statistical Analysis

The statistical analysis of the preliminary tests and cadaver studies was carried out with the software SPSS 20 (SPSS Science, Chicago, IL, USA). For the tube test, the dependence of the measurement results on the distance from the endoscope to the object as well as the location and number of reference points was plotted using a scatter plot. In addition, the mean deviation of the measurements from the set point value was determined for all 100 consecutive measurements at a distance of 5 cm from the object. In addition, the Spearman correlation was determined for the laryngeal and trachea measurements. A Bland-Altman-Plot was used to calculate the 95% limits of agreement to compare the method with the standard measurement. A nominal *p* value < 0.05 was considered to be statistically significant.

## 3. Results

**Figure 6:**
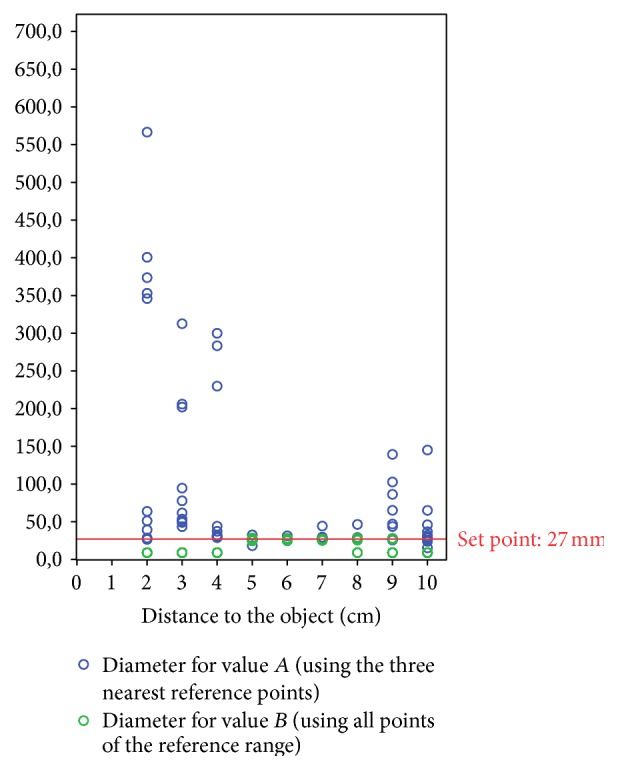
Dependency of the measured diameter in relation to the distance of the endoscope tip to the object (values *A* and *B*).

**Figure 7:**
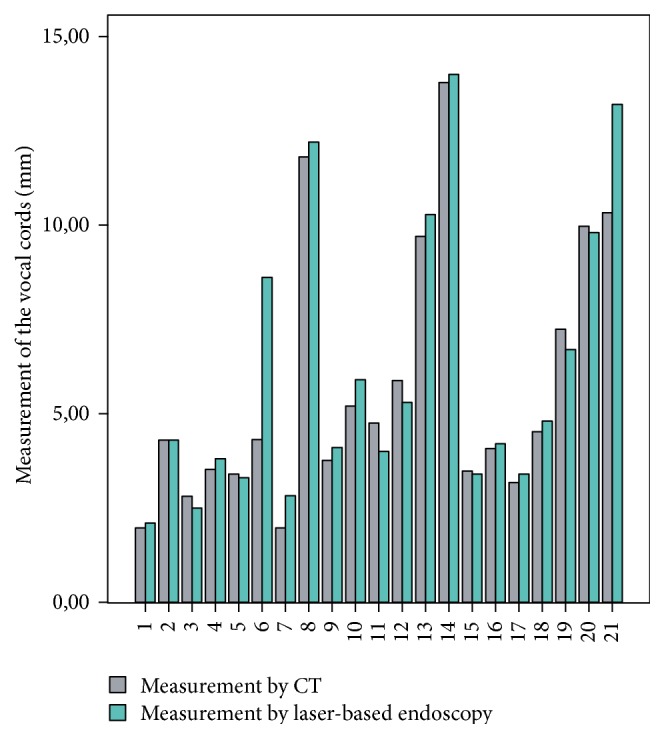
Comparison of the laryngeal measurements by laser endoscopy compared to CT (*r* = 0.95, *p* = 0.01).

**Figure 8:**
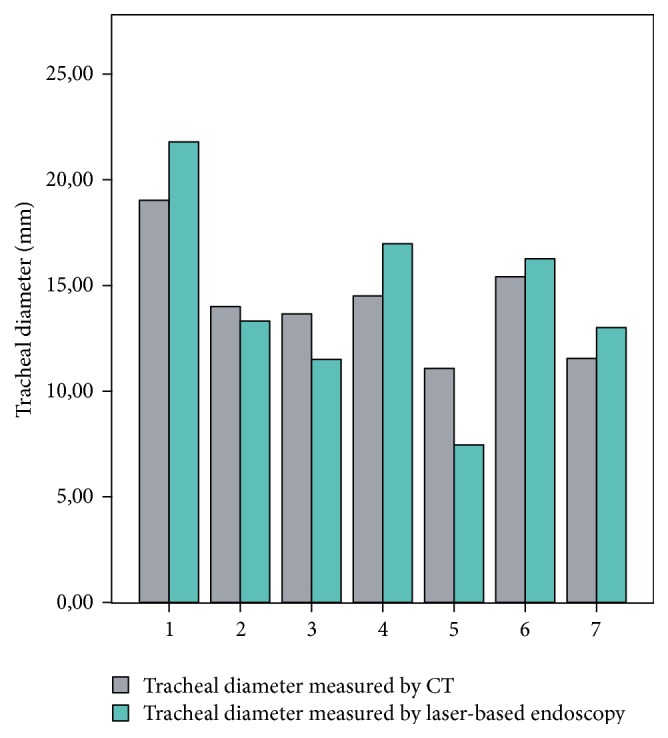
Comparison of the tracheal measurements by laser endoscopy compared to CT (*r* = 0.93, *p* < 0.01).

The tube experiment showed that the optimum distance between the endoscope tip and the object to be measured is between 5 to 6 cm (Figure 6): mean 26.38 mm  ±  0.84 mm [range: 24.17 mm–28.78 mm] and mean deviation error 3.26%  ±  2.53%. Distance measurements with reference points whose *z*-coordinates are located in the same plane (±5 mm) as the object under test showed significantly better results than measurements with reference points outside this range: 26.33 mm versus 73.28 mm, *p* < 0.01 (Table 1). An average of 8.4 ± 1.7 [range: 4–13] recognized laser beam points was found. However, the number of recognized laser beam points found showed no significant relevance for the measurement results: Pearson correlation coefficient of −0.063 (*p* = 0.536). A Spearman correlation coefficient of 0.95 (*p* = 0.01) was calculated for the laryngeal measurements compared to the CT (Figure 7). For the tracheal measurements the Spearman correlation coefficient was 0.93 (*p* < 0.01) compared to the CT (Figure 8). Using the Bland-Altman-Plot, the 95% limits of agreement for the laryngeal measurements were −0.76 and 0.93 and for the tracheal measurements were −4.65 and 4.95.

## 4. Discussion

Minimally invasive techniques and endoscopic surgical approaches are becoming increasingly important for ENT surgery. For instance, the standard procedure for the diagnostic workup and clinical staging of HNSCC is the upper airway endoscopy under general anesthesia with precise description of the tumor extent. Similarly, the rigid endoscopy is considered the technique of choice in the diagnosis of airway stenoses. Technological advances, particularly in diagnostic endoscopy (HD image quality, stereo endoscopy), result in a more precise use of these techniques. Furthermore, the accessibility and visualization of robotic (endoscopic) surgery systems have been improved considerably. Hence, the use of transoral robotic and endoscopic surgery extends beyond the oropharynx into the larynx and hypopharynx [18, 19].

In the area of the oral cavity and the upper oropharynx the sizes of lesions can be measured using conventional measuring instruments. However, the sizes of lesions in the area of the tongue base, the larynx, or hypopharynx or the trachea are usually estimated by endoscopic inspection. The continuous selection of the focus, the magnification, and the endoscope distance to the region of interest are confounding factors that distort the object measurement based on the optical image. Mostly, endoscopic measuring methods are based on the method of comparative measurement. An object of a known size is compared directly with the lesion or the cross-section to be measured in the endoscopic view. However, significant underestimations of lesion sizes by endoscopic inspection are known [22, 23]. Although this measurement method can be easily performed with inexpensive efforts, there are still some disadvantages, for example, risk of injury by using such a measuring device, and it can be used for rigid endoscopy only. Furthermore, the measurement results are still not satisfactory: Sharma et al. published rates of measurement agreement of 82.5% for the subglottic diameter and of 72.5% for the measured lengths [2].

Image postprocessing using software algorithms which automatically corrects image distortion of the optical image is another method to improve endoscopic measurement. However, a calibration of the hardware and software is still necessary [24]. The reference bodies with scales between predefined points have to be touched in a coplanar fashion against the object to be measured. If the reference body is not at the exact same planar level measurement accuracy is affected [3]. Furthermore, regularly no real-time measurement is possible [25]. However, postprocessing algorithms have been described in gastrointestinal endoscopy that can be performed faster compared to traditional measurement [4, 5].

3D visualization with stereo endoscopes allows shape reconstruction with measurement. However, endoscopes have to be calibrated: feature points have to be detected and corresponding distances have to be determined with a robust estimation of the camera motion, especially in the *z*-plane [6, 7]. So far scientific approaches have been published which, however, are lacking commercially available integrated measurement systems.

This study describes the use of a fully integrated multipoint-laser endoscopic measurement system by transoral approach. The commercially available system allows real-time measurements during endoscopy of the upper airways. The validation experiment showed an optimal distance of the endoscope tip of 5 to 6 cm to the object to be measured. If the recognized laser beam points were in the measurement plane, the highest measuring accuracy was achieved with a mean deviation error of 3.26%. No correlation in the measurement accuracy could be shown depending on the number of additionally recognized laser beam points. The comparison between multipoint-laser endoscopic measurements of the upper airway and CT measurements showed correlation coefficients for the larynx (*r* = 0.95, *p* < 0.01) and the trachea (*r* = 0.93, *p* < 0.01). Satisfactory 95% limits of agreement were calculated for the laryngeal measurements; unfortunately the 95% limits of agreement were wider for the tracheal measurements.

Advantages of this fully integrated “stand-alone” system are that there is no need for calibration, and a noncontact measurement functionality using real-time endoscopic imaging is provided. In particular, the edge and center emphasizing function is an excellent condition for the measurement of tubular objects. This would allow its use in TORS, with a straight view to the oropharyngeal and laryngohypopharyngeal level. The determined optimum distance of 5 to 6 cm corresponds well to the use of endoscopy in TORS by different robotic systems [18–20]. For example, the nominal working distance of a stereo endoscope used in the da Vinci telemanipulator is about 4 cm [26]. Furthermore, Nakano et al. showed accurate and sufficiently sized images positioning at the tip of a stereo endoscope at a distance from 2.0 to 6.5 cm from the object for endoscopic analysis of velopharyngeal movement [8].

Not only the measurement of distances but also the measurement of cross-sectional areas and depths is a special feature compared to previously described systems. Thus, it is also suitable for the measurement of lesions in tubular organs. Therefore, the description of the longitudinal and cross-sectional dimension of filiform or pinhole tracheal stenoses would be possible. Referring to the accuracy of the measurements, the study showed comparable results to data from the literature. A correlation coefficient higher than 0.9 and a mean measurement error of 3.26% were only achieved by a few systems. Dörffel et al. achieved a correlation of 0.88 with measurements on pig tracheas [15]. Müller reports a measurement error of less than 5% for tracheal measurements [16]. These systems have in common that a laser fiber is inserted into a flexible endoscope for circular scanning of a tubular object with laser beam projection. However, the depth of the measurement (*z*-axis) was determined by manual measurement of the push-forward distance. Other laser measurement systems used in gastrointestinal endoscopy showed measurement errors of 3.7%–6.5% with laser diffraction, 5.1% with four laser beams, and, respectively, 4.0% with two lasers and a virtual grid [1, 27, 28]. However, the 95% limits of agreement vary widely for the tracheal measurements. This might be explained by underestimated CT measurements due to fluid accumulation in the tracheal specimens. Although the 3D shape reconstruction methods using photometric stereo may provide objective size and object determination of endoscopic images, only experimental data based on computer simulations and single gastrointestinal endoscopy images have been described so far. Despite ideal reflection and recording parameters and experimental ranges of the endoscope movement (*z* = 3 mm) the processing times exceed 2 minutes limiting its use for daily routine practice [11]. For regular endoscope use its movement has to be tracked for determination of the *z*-coordinate. This would require further technical supplement limiting its applicability. In addition, the measurement errors are still higher compared to the system described in this study (9.1% versus 3.26%; 7.8–12.5% versus 3.26%) [12, 29].

Not only the results of the accuracy but also the setup of the multipoint-laser measurement systems offers advantages compared to previously described systems. The diameter of less than 5 mm allows its use for upper airway evaluation without imposing a burden on the patient. Additionally, it can be used safely for endoscopic real-time measurements. Laser beam points in the same plane are used for distance and area measurements; laser beam points reflected from objects in different planes are used for depths measurements. The 7 by 7 grid of laser beam points allows the specific selection of laser beam points in the complex anatomical area of the pharynx, larynx, and trachea which are difficult to measure due to their anatomical complexity [3]. Thus, it is a fully integrated endoscope unit, without the need for additional technical supplements or measuring devices to be introduced. Therefore, the system can be used without need for calibration or image-processing.

A confounding factor, which is reported in the literature, is the increase in the mean measurement error with an increasing tilt angle of the endoscope tip. In this study a semirigid endoscope with a straight view was used with perpendicular visualization of the larynx and trachea by transoral approach. However, the system specification indicates a maximum increase of 2% of the measurement error at 30° tilt angle of the endoscope. Due to the fact that this system would be available with a flexible endoscope, too, it is suitable for the transoral approach in TORS, as a perpendicular view can always be ensured. Although the system offers many advantages, a typical problem of laser-based endoscopic measurement is not solved. These are reflection highlights on glossy mucosal surfaces, which impede the automatic detection of the laser beam points. This has not been described for laser-based measurement only, but for 3D endoscopic laparoscopic measurement, too [1, 29]. A reduced intensity of the illumination helps to solve this problem.

## 5. Conclusion

Integrated multipoint-laser endoscopic measurement is a promising technical supplement, with potential use in diagnostic endoscopy and transoral endoscopic surgery in daily practice. In particular, for TLM and TORS, this tool is an option for the exact preoperative measurement of a lesion or the exact description of the extent of the surgical resection area. Other applications include diagnostic endoscopy evaluation of tracheal stenoses or macroscopic determination of tumor surface reduction during response evaluation to induction chemotherapy.

## Figures and Tables

**Table 1:** Distance measurements depending on the distance object-endoscope tip.

Distance object-endoscope tip	All recognized laser beam points	Value *A*	Value *B*	Deviation value *A* in %	Deviation value *B* in %
2 cm	0	224,91		60,9	
3 cm	0	115,19		326,6	
4 cm	0	104,59		287,4	
5 cm	7,9	25,9	26,13	8,1	4,4
6 cm	7,1	26,44	26,01	5,3	4,6
7 cm	5,3	28,86	26,18	13,4	3,1
8 cm	3,2	29,46	26,94	12,57	2
9 cm	0,7	59,12	27,35	120,6	2
10 cm	0	45,01		78,2	
